# Affective Variables and Cognitive Performances During Exercise in a Group of Adults With Type 2 Diabetes Mellitus

**DOI:** 10.3389/fpsyg.2020.611558

**Published:** 2020-12-23

**Authors:** Marco Guicciardi, Daniela Fadda, Rachele Fanari, Azzurra Doneddu, Antonio Crisafulli

**Affiliations:** ^1^Department of Education, Psychology, Philosophy, University of Cagliari, Cagliari, Italy; ^2^Sports Physiology Laboratory, University of Cagliari, Cagliari, Italy

**Keywords:** type 2 diabetes mellitus, exercise, cognitive impairment, near-infrared spectroscopy, attentional task

## Abstract

Previous research has documented that type 2 diabetes mellitus (T2DM) is associated with cognitive impairment. Psychological variables were repeatedly investigated to understand why T2DM patients are poorly active, despite standards of medical care recommends performing aerobic and resistance exercise regularly and reducing the amount of time spent sitting. This exploratory study aims to investigate how affective variables as thoughts, feelings, and individuals’ stage of exercise adoption can modulate low cognitive performances during an experimental procedure based on exercise. The Exercise Thoughts Questionnaire (ETQ), Exercise-Induced Feeling Scale (EFI), and Physical Activity Stage of Change were administered to a sample of 12 T2DM patients. The Bivalent Shape Task (BST) alone (BST), BST with exercise [control exercise recovery (CER) + BST], and BST with metaboreflex [post-exercise muscle ischemia (PEMI) + BST] were used as mental task, and response time to congruent, incongruent, and neutral stimuli was recorded. Concomitant cerebral oxygenation (COX) was evaluated by near-infrared spectroscopy (NIRS). As expected, T2DM patients performed significantly better when the stimulus was presented in congruent trials (followed by neutral and incongruent). In the CER + BST session, T2DM patients showed longer reaction time to incongruent trials than in the PEMI + BST and BST alone sessions. Positive feelings toward exercise seem to modulate cognitive performances in high challenging task only if T2DM patients were conscious to play exercise. These results could provide some insights for health intervention targeting exercise for patients with T2DM in order to enhance cognitive performances.

## Introduction

Diabetes is one of the fastest growing health challenges of the 21st century: the number of adults living with diabetes, estimated currently equal to 463 million, having more than tripled over the past 20 years. Type 2 diabetes mellitus (T2DM) corresponds to the most prevalent type of diabetes in the world: its increasing rate is connected to growing urbanization and changing lifestyle habits (International Diabetes Federation, 2017).

Regular exercise, together with a healthy diet, represents the most important preventive factor for people at risk of type 2 diabetes. Exercise improves insulin resistance and blood glucose control, increases balance and coordination, enhances brain plasticity, cardiorespiratory endurance, and well-being. Exercise has beneficial effects on the control of many factors related to diabetes (lipid profile, hypertension, and obesity; Tuomiletho et al., 2001; Lunghi et al., 2019a) and allows to reduce medication number and/or dosage (Balducci et al., 2012). The recommendations of the International Diabetes Federation (IDF) for managing type 2 diabetes encourage intensive therapeutic lifestyle changes such as exercise, the reduction of cholesterol, and the dietary intake of saturated fat (Aschner, 2017). More specifically, moderate aerobic physical activity such as walking for at least 150 min per week at intervals of no longer than 48 h was recommended. Resistance exercise such as moderate weightlifting or yoga can also be included. A more intensive physical activity program including at least 275 min per week may be needed to assist weight loss and avoid regaining it (International Diabetes Federation, 2017).

Although the literature is very large and the benefits of exercise are now well known (Lunghi and Sale, 2015; Tomas-Carus et al., 2016; Podolski et al., 2017; Lunghi et al., 2019b), inconsistent findings were sometimes reported (Zhao et al., 2018; Cooke et al., 2020; Dyer et al., 2020).

Exercise can influence cognitive functions by increasing brain activation and cerebral blood flow and perfusion (Rooks et al., 2010; Dupuy et al., 2015). Exercise increases (on average of one-half standard deviation) cognitive performance, independently of the cognitive task, the characteristics of participants, and the training method (Colcombe and Kramer, 2003). Executive function enhancement was reported after 6 months of aerobic training (Baker et al., 2010), while functional plasticity of response inhibition process improved after 12 months of resistance training (Liu-Ambrose et al., 2012). The engagement in simultaneous exercise and cognitive training (dual-task training) has been shown to improve cognition beyond the effects of the single underlying components (Cooke et al., 2020).

T2DM patients show an incidence double of cognitive impairment compared to non-diabetic adults (Lyu et al., 2020). In several cognitive domains as attention, processing speed, visuospatial abilities, memory, executive functions, and semantic fluency, poor performances in T2DM patients were often reported (van den Berg et al., 2009; Palta et al., 2014; Fava et al., 2017; Zhao et al., 2018; Sun et al., 2020). Neuropsychological cross-sectional studies (Manschot et al., 2007; Brundel et al., 2010) and longitudinal studies (van Elderen et al., 2010) found brain atrophy and white matter loss in patients with T2DM. More recently, systematic reviews and meta-analyses were also conducted, confirming these findings (cfr. Sadanand et al., 2016; Zhao et al., 2018; Cooke et al., 2020). Despite the increasing evidence, to date, the exact underlying mechanisms explaining cognitive dysfunction in T2DM remain unclear.

Exercise has been supposed to improve cognition by different mechanisms, directly or indirectly connected to glucose metabolism control, as increased synaptogenesis and neurogenesis, enhanced cerebral perfusion, reduced inflammation, increased availability of neurotrophins and neurotransmitters, and reduced cerebral atrophy (Tomas-Carus et al., 2016; Cooke et al., 2020). Thus, exercise may be useful to improve cognitive processes as executive functions and to reduce cognitive impairment (Secher et al., 2008; Baker et al., 2010; Kim et al., 2011; Zhao et al., 2020); however, in T2DM patients, impaired cerebral blood flow and oxygenation during exercise were reported (Kim et al., 2015; Vianna et al., 2015), producing relevant costs in addition to the well-known benefits.

A similar pattern was also observed in response to stimulation of metaboreflex (Delaney et al., 2010; Crisafulli, 2017) generated by metabolites accumulating in the muscle during contraction (Boushel, 2010). Muscle metaboreflex activated by metabolites accumulating in the muscle during contraction can enhance similarly the reduction of cerebral oxygenation (COX) and can impair cognitive functions and produce early fatigue (González-Alonso et al., 2004; Rasmussen et al., 2010). Therefore, in T2DM patients, the overlapping of a high-demand cognitive task to exercise could undermine the optimal neuronal environment. In a previous study conducted with patients suffering from metabolic syndrome (MS), we have observed that the connection between a concurrent mental task and metaboreflex can hesitate in a reduction of COX and in a deterioration of cognitive performance (Guicciardi et al., 2019; Doneddu et al., 2020).

Moving from these premises, we want to explore the contribution of affective states in their interplay with exercise, metaboreflex, and cognitive performance in a group of T2DM patients. Affective responses to exercise, as positive feelings or negative thoughts, were already investigated in T2DM patients, mainly to explain low adherence and retention rates to exercise (Guicciardi et al., 2014a) or to assess the feasibility and efficacy of specific exercise programs, as high-intensity interval training (Terada et al., 2013). However, more recent studies pointed out that affective states can be considered as regulators of exercise performance (Hartman et al., 2019).

Since the results obtained in the laboratory are often not easily generalizable to everyday life, we have also considered the assessment of the stage of exercise adoption, which has been proven to be a useful tool to target exercise interventions in T2DM patients (Kim et al., 2004; Kirk et al., 2010). Namely, we contrasted the two stages of preparation and action, which were considered critical to adopt an active lifestyle by individuals suffering from T2DM (Guicciardi et al., 2014b).

Thus, the present study intends: (a) to extend previous findings obtained with MS patients to a sample of T2DM people and (b) to investigate in an explorative way the contribution of affective variables (i.e., adoption, feelings, and thoughts toward exercise) as moderators of the relationship between cognitive performance and exercise in a group of T2DM patients.

## Materials and Methods

### Participants

A group of 12 T2DM patients was enrolled [five women, mean ± standard deviation (SD) of age 49.5 ± 10.0 years] on the basis of the following criteria: clinical history of T2DM for at least 1 year (range 1–6 years), stable metabolic condition (HbA1c level <9% at the time of the study), and absence of signs or symptoms of peripheral neuropathy. All patients were on medication with oral hypoglycemic agents; 11 with insulin (Table 1).

**Table 1:** Descriptive statistics of the sample.

Variables (range)	*Mean*	*SD*
Age	49.50	10
BMI	30.54	5.78
Blood pressure (max)	123.75	14.94
Blood pressure (min)	82.08	10.54
Plasma glucose	106.58	12.96
HbA1c	6.42	0.73
Duration of diabetes (years since diagnosis)	3.17	1.58

BMI, body mass index; HbA1c, glycated haemoglobin.

### Instruments

The Exercise Thoughts Questionnaire (ETQ; Kendzierski and Johnson, 1993) measures by 25 items how frequently exercisers have exercise avoidant thoughts. Participants respond to a 5-point Likert scale with 5 anchored by “all the time” and 1 anchored by “not at all.” Typical items include “I have not got time,” “I’ll do it tomorrow,” and “I’m too busy.” This instrument was already used to assess negative thoughts toward exercise in T2DM patients (Guicciardi et al., 2014a).

The Exercise-Induced Feeling Inventory (EFI; Gauvin & Rejeski, 1993) measures by 12 adjectives four feeling states: revitalization, tranquility, positive engagement, and physical exhaustion. The items are rated on a 5-point scale from 0 to 4, where 0 stands for “do not feel at all” and 4 stands for “feel very strongly.” This instrument was already used to assess positive feelings toward exercise in people at risk for T2DM (Masters et al., 2011).

The stage of exercise adoption (SEA) was assessed by asking participants to choose which of five statements, each representing a stage of change, described their current exercise commitment (Marcus et al., 1992). The stages of change can be distinct on: precontemplation (not regularly physically active and no thought to become active in the next 6 months); contemplation (not regularly physically active but aiming to start in the next 6 months); preparation (doing some physical activity but not enough to meet the description of regular physical activity); action (regularly physically active but only begun in the last 6 months); and maintenance (regularly physically active for more than 6 months). This instrument was already used to assess exercise adoption in T2DM patients (Kirk et al., 2010; Guicciardi et al., 2014b).

The Bivalent Shape Task (BST; Esposito et al., 2013) is a simple and fast nonverbal measure of cognitive interference and suppression that requires the participant to determine whether a shape at the center of the screen is a square or a circle. Two response targets are provided below the stimulus, one shaped as a circle and one as a square. The target circle is always on the left, and the square is always on the right. The participant, equipped with a mouse, is asked to click the response target corresponding to the center of the screen stimulus shape, ignoring stimulus and target color. The stimulus shape is presented in red, blue, or an unfilled black outline; response targets can be presented in red or blue. Three trial types exist: neutral (black or white stimulus); congruent (the stimulus color matches the response target color); incongruent (the stimulus color mismatches the response target color). The task was performed using an open source programming language (cross-platform) created to implement psychological tests. The response times were recorded in ms. The BST was already used as a mental task in a group of adults suffering from MS (Guicciardi et al., 2019).

The near-infrared spectroscopy (NIRS; Nonin, SenSmart X-100, Plymouth, MN, USA) was used to assess COX, providing a measure of oxygenated hemoglobin (Hb) in the brain tissue. NIRS was already used to assess COX during mental tasks (e.g., calculation), BST interference tasks, or Stroop tests in the general population (Plichta et al., 2006; Ferreri et al., 2014) and in MS patients (Guicciardi et al., 2019; Doneddu et al., 2020). Researchers placed two NIRS sensors in the subject’s right and left sides of the forehead above the eyebrow (between Fp1 and F3 regions, international EEG 10–20 system) and adjusted according to the stronger signal. COX variations are representative of cortical activation (Strangman et al., 2002). Researchers considered the relative changes of NIRS signals vs. the baseline values; indeed, the absolute concentration of Hb cannot be obtained, since the path length of NIRS light within the brain tissue was unknown.

### Procedure

All participants, after a medical examination, were assigned in a random order to five sessions. All sessions lasting 12 min were composed of four blocks (3 min per block) spaced by a recovery of 15 min (cfr. Guicciardi et al., 2019, and Doneddu et al., 2020, for more details):

- BST session comprises a rest period of 6 min, 3 min of mental task and 3 min of further recovery.
- Control exercise recovery (CER) session comprises a rest period of 3 min, 3 min of rhythmic (i.e., 30 compressions/min) dynamic handgrips in the nondominant hand using a dynamometer, a rest period of 3 min.
- Post-exercise muscle ischemia (PEMI) session comprises 3 min of resting, followed by 3 min of exercise, as in the CER session, followed by 3 min of PEMI on the exercised arm induced by rapidly inflating an upper arm biceps tourniquet to 50 mmHg above peak exercise systolic pressure. Three minutes of recovery was further allowed after the cuff was deflated, for a total of 6 min of recovery. This maneuver has been demonstrated to be capable of eliciting the metaboreflex-induced hemodynamic stimulation and able to detect cardiovascular abnormalities (Crisafulli, 2017).
- CER + BST session comprises a rest-exercise protocol (the same used for CER). The exercise phase was followed by a BST session and a recovery period each for 3 min.
- PEMI + BST session comprises the rest-exercise protocol utilized for PEMI, followed by a block including both PEMI and BST. Finally, the session was concluded with 3 min of recovery.

This study was designed according to the recommendations of the Code of Ethics for Research in Psychology, Italian Association of Psychology. The protocol was approved by the ethics committee of the University of Cagliari. All subjects gave written informed consent in accordance with the Declaration of Helsinki.

### Data Analysis

Only data relating to sessions where BST was included were analyzed (BST, CER + BST, PEMI + BST). A preliminary check of data was executed (Kolmogorov-Smirnov test) to determine whether variables were normally distributed. After controlling for age and body mass index (BMI), repeated measures analyses were carried out to assess: (a) response times (ms) on BST and (b) change in COX (% from rest). Further explorative analyses were conducted on affective response test scores using a repeated measures ANCOVA with the individual’s stage of exercise adoption as an independent variable and the positive feeling state as a covariate.

Statistics were carried out utilizing SPSS ver. 24.0. A value of *p* < 0.05 (statistical significance) was set up in all cases.

## Results

Descriptive statistics are reported in Table 1.

After controlling for age [*F*_(2,20)_ = 2.266, *p* = 0.130, eta squared = 0.185] and BMI [*F*_(2,20)_ = 0.547, *p* = 0.587, eta squared = 0.052] through two ANCOVAs, repeated measures analyses showed that, overall, the three sessions with the BST (PEMI + BST, CER + BST, and BST) were not associated with differences on response times [*F*_(2,22)_ = 1.715, *p* = 0.203, eta squared = 0.135; Table 2a].

**Table 2:** Response time mean and standard deviation.

		*Mean*	*SD*
a)	BST	853.19	133.77
	CER + BST	890.63	177.03
	PEMI + BST	861.17	178.86
b)	Congruent	843.18	155.76
	Neutral	872.67	148.43
	Incongruent	889.14	174.63
c)	BST Congruent	819.14	138.16
	CER + BST Congruent	867.43	181.16
	PEMI + BST Congruent	842.98	165.69
	BST Neutral	864.21	123.16
	CER + BST Neutral	887.03	176.91
	PEMI + BST Neutral	866.77	161.88
	BST Incongruent	876.57	150.48
	CER + BST Incongruent	917.42	181.34
	PEMI + BST Incongruent	873.77	213.27

BST, bivalent shape task; CER, control exercise recovery; PEMI, post-exercise muscle ischemia.

As for the BST performance, as expected, results showed a main effect of the type of trial variable, showing that participants performed significantly faster when the stimulus was presented in congruent trials, followed by neutral and incongruent ones [*F*_(2,22)_ = 12.951, *p* = 0.000, eta squared = 0.541] (Table 2b).

**Figure 1:**
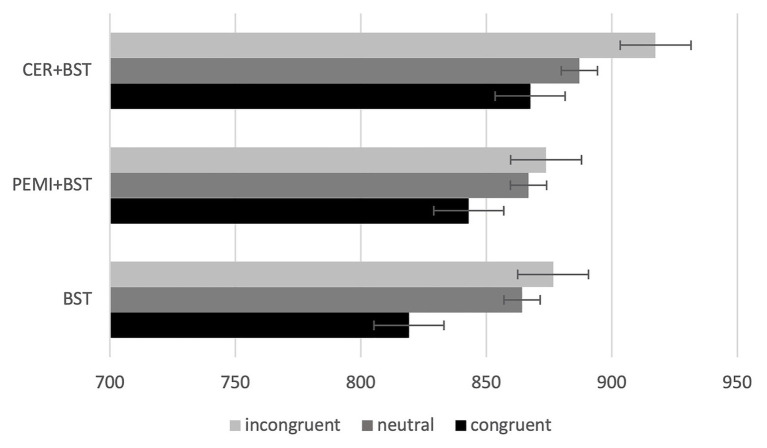
Mean response time and standard error (bars) by sessions and trials. BST, bivalent shape task; CER, control exercise recovery; PEMI, post-exercise muscle ischemia.

Moreover, only CER + BST [*F*_(2,22)_ = 5.102, *p* = 0.015, eta = 0.317] and BST [*F*_(1.318,22)_ = 6.837, *p* = 0.140, eta = 0.383] showed significant differences between congruent, neutral, and incongruent stimuli (Figure 1). Marginal mean comparisons with Sidak adjustment showed differences associated with congruent and incongruent stimuli for CER + BST (*p* = 0.013) and BST (*p* = 0.011) sessions and with neutral and congruent stimuli for BST (*p* = 0.004; Table 2c).

The COX expressed as the cerebral oxygenation percentage variation from rest level significantly increased during the third minute of all sessions. *Post hoc* analysis revealed that CER + BST (*M* = 103.10; *SD* = 2.89) and post-PEMI + BST (*M* = 102.24; *SD* = 2.00) showed more increases (*p* > 0.05) compared to BST session alone (*M* = 100.36; *SD* = 1.10).

To better understand these differences, the individual’s stage in the exercise adoption (dichotomized in preparation/action) was added into the analysis, together with the positive feelings and the negative thoughts toward exercise. A correlational analysis (Spearman’s rho coefficient) among all affective response scores showed a significant negative relationship between negative thoughts to exercise and stage in exercise adoption (Table 3).

**Table 3:** Correlations among psychological variables.

	EFI	SEA	ETQ
EFI	1		
SEA	0.025	1	
ETQ	−0.444	−0.590^*^	1

EFI, Exercise-induced feeling scale; SEA, stage in exercise adoption; ETQ, exercise thoughts questionnaire.

* *p* < 0.05.

After controlling with Mauchly’s test that the assumption of sphericity had not been violated [*χ*^2^_(2)_ = 0.911, *p* = 0.634], an ANCOVA was performed using stage of exercise adoption as a group factor, response time at sessions as a repeated dependent variable, and positive feeling about exercise as a covariate. In relation to CER + BST session, a significant interaction effect did not emerge between the stage of exercise adoption and congruence, but the positive feelings were a significant covariate [*F*_(2,18)_ = 3.714, *p* = 0.045, eta = 0.292], suggesting that this affective variable represents a source of variability that might have an effect on the outcome.

## Discussion

The ability to exercise regularly is critical for functional independence and well-being of adults suffering from T2DM. Hence, identifying factors that influence exercise adoption and cognitive impairment among adults with diabetes has important clinical and public health implications.

Executive functions are necessary for behavior change and may have the potential to affect an individual’s capability to successfully adopt and maintain exercise (Olson et al., 2017). Successful management of diabetes is determined by the implementation of strategies able to mitigate the impairment of executive functions (Zhao et al., 2020).

Exercise appears to be a useful tool for T2DM patients because it reduces cognitive impairment and diabetes complications. However, exercise adoption requires more time and deliberate effort than just diet modification or medications taking, and it is perceived as a difficult and significant modification of the way of life (Guicciardi et al., 2014a). Thus, is imperative to understand how to support these changes (Conversano, 2019) by identifying the psychological variables that facilitate exercise adoption and act as protective factors toward cognitive impairment.

The main aims of the present study were: (a) to depict the cognitive performance in T2DM patients during exercise, with the association of a mental task (BST) to metaboreflex activated by means of the PEMI and (b) to extend the understanding of cognitive impairment during exercise in a group of T2DM people, introducing affective variables as feelings and thoughts toward exercise and the individual’s stage in the exercise adoption.

With regard to cognitive performance, the mean response time did not differentiate the three sessions where a mental task was imposed. As expected, the T2DM patients, as well as the general population (Esposito et al., 2013), performed better with congruent trials compared to neutral and incongruent ones. However, when the interaction between type of sessions and type of BST trials was observed, T2DM patients performed worse in the CER + BST session, showing a linear increase of response times. These results are consistent with the neural inefficiency hypothesis (Zarahn et al., 2007), wherein higher brain activation is associated with worse task performance, suggesting that T2DM patients do not efficiently allocate cognitive resources to support high demanding mental tasks. Indeed, incongruent trials require participants to suppress the interferent immediate response to the stimulus color and consider only the relevant information (stimulus shape) to give the correct response. This interference does not appear in the PEMI + BST session and in the BST alone session, where the mean response time for incongruent stimuli was equal to neutral ones. Similar findings were also reported by Holtzer et al. (2018) using a dual-task walking with T2DM older adults.

The capacity to suppress the immediate response is crucial to manage T2DM because the acquisition of new behavior requires the inhibition of a habit (e.g., sitting after meals) and replacement with a healthier behavior (i.e., going for a walk; Settineri et al., 2019).

This impaired capacity is more amazing because CER + BST and PEMI + BST sessions showed a similar increase of COX compared to BST alone, confirming that both implicate a metabolic expenditure involving equivalent levels of sympathetic tone (Crisafulli, 2017).

Feelings about exercise seem to offer a supplemental explication of this phenomenon because only in the CER + BST session do positive feelings affect mean response times. Ratings of pleasure-displeasure were already used as indicators of the severity of the homeostatic perturbation during exhaustive exercise (Hartman et al., 2019). Studies conducted in the healthy population indicate that changes along the dimension of pleasure-displeasure can be considered the main channel by means of homeostatic perturbations entering consciousness and dictating corrective action, as slowing down or stopping exercise (Damasio and Carvalho, 2013). However, to date, to the best of our knowledge, this is the first time that affective responses and cognitive performances were jointly investigated during exercise in T2DM patients. The different contributions of positive feelings to cognitive performance claim for a deeper analysis of affective process and open new perspectives on exercise prescription and self-care in T2DM patients (see Martino et al., 2020, for a similar suggestion).

Although structured exercise interventions have been shown to be effective in increasing physical activity levels and improving cardiovascular fitness and glycemic control during the intervention period, there is little evidence that these strategies have effects in the long run, if people are not supported in changing their habits. However, to support some form of lasting change, it is necessary to know what mechanism can counteract the emotional stress experienced by T2DM patients during exercise (Armstrong and Sigal, 2015; Guicciardi et al., 2015) and in the course of daily life (Tomas-Carus et al., 2016; Martino et al., 2019). Positive feelings appear to play a protective role against the distress caused by exercise in T2DM patients who are performing, in an experimentally controlled condition, an attentional interference task, but further studies are needed to assess their contribution in everyday life.

Data related to the effects of experimental sessions on response times were examined and used to infer information about the brain mechanisms associated with mental task while conducting an exercise. The different mean response times for the congruent and incongruent stimuli in two equivalent conditions of metabolic expenditure (CER + BST and PEMI + BST) should be emphasized: while in the PEMI + BST, there is not a significant difference between congruent and incongruent trials’ response times, in the CER + BST, the incongruent trials are answered slower than the congruent ones. It seems that participants have experienced the difficulty of the coupling of exercise and cognitive demand more in a condition than in the other. While CER + BST is an explicit condition of physical exercise, the PEMI + BST emulates the metabolic expenditure produced by exercise by means of the enhancement of the sympathetic tone and sympathetic nervous system (SNS) activity (Boushel, 2010). A possible explanation of our findings is that the disadvantage of the most demanding trial condition (the incongruent one) becomes more evident when an exercise is explicitly required. Moreover, it is noteworthy that the contribution of positive feelings, as already noted, appears only in the CER + BST session as if the affective response to exercise manifests itself only when the brain predicts that some perturbation will change the general homeostasis. This tentative hypothesis is further supported by findings that the ratings of perceived exertion are mostly unrelated to indices of metabolic strain (e.g., heart rate, blood lactate, and respiratory frequency) at low intensities (Hartman et al., 2019). The results of our exploratory study are promising, but the small size of our sample does not authorize more speculations. More studies, as randomized controlled trials and large-scale studies, also looking for gender differences and age-related effects, should be developed to investigate the mechanisms that regulate the relationships between affective response to exercise, consciousness, COX, and cognitive performances in T2DM patients.

### Limitations of the Study

The study suffers some limitations. This was a cross-sectional study with a small sample size and without a randomized control group. However, this study extends a previous one conducted with an MS group and a matched control group, in which the same experimental setting was used. The indirect comparison between our consecutive studies dampens but does not eliminate this flaw. The BST was already used with MS patients, but further studies are necessary to validate this task with T2DM patients. NIRS, compared to others neuroimaging techniques, suffers from low spatial resolution, but its sensitivity to measure subtle changes in COX in the normal population and MS patients during cognitive tasks and exercise already has been proven.

In conclusion, patients with T2DM achieve worse cognitive performance when incongruent stimuli were presented during exercise, but not when the same stimuli were presented in a condition of equivalent metabolic expenditure. Moreover, positive feelings about exercise seem to modulate cognitive performance in T2DM patients in response to high requiring stimuli, only when an attentional task was associated with a deliberate practice.

## Data Availability Statement

The raw data supporting the conclusions of this article will be made available by the authors, without undue reservation.

## Ethics Statement

The studies involving human participants were reviewed and approved by Ethics Committee of the University of Cagliari. The patients/participants provided their written informed consent to participate in this study.

## Author Contributions

The original study design was made by AC and MG and was discussed with the other authors. AD and DF performed the experiments and collected and analyzed, respectively, functional and psychological data. RF conducted the formal analysis. MG and AC wrote, reviewed, and edited the manuscript. All authors contributed to the article and approved the submitted version.

### Conflict of Interest

The authors declare that the research was conducted in the absence of any commercial or financial relationships that could be construed as a potential conflict of interest.

## References

[ref1] Armstrong M. J. Sigal R. J. (2015). Exercise as medicine: key concepts in discussing physical activity with patients who have type 2 diabetes. Can. J. Diabetes 39, S129–S133. 10.1016/j.jcjd.2015.09.081, PMID: 26653253

[ref2] Aschner P. (2017). New IDF clinical practice recommendations for managing type 2 diabetes in primary care. Diabetes Res. Clin. Pract. 132, 169–170. 10.1016/j.diabres.2017.09.002, PMID: 28962686

[ref3] Baker L. D. Frank L. L. Foster-Schubert K. Green P. S. Wilkinson C. W. McTiernan A.. (2010). Effects of aerobic exercise on mild cognitive impairment: a controlled trial. Arch. Neurol. 67, 71–79. 10.1001/archneurol.2009.307, PMID: 20065132 PMC3056436

[ref4] Balducci S. Zanuso S. Cardelli P. Salvi L. Bazuro A. Pugliese L.. (2012). Effect of high‐ versus low-intensity supervised aerobic and resistance training on modifiable cardiovascular risk factors in type 2 diabetes; the Italian Diabetes and Exercise Study (IDES). PLoS One 7:e49297. 10.1371/journal.pone.0049297, PMID: 23185314 PMC3504024

[ref5] Boushel R. (2010). Muscle metaboreflex control of the circulation during exercise. Acta Physiol. 199, 367–383. 10.1111/j.1748-1716.2010.02133.x, PMID: 20353495

[ref6] Brundel M. van den Heuvel M. de Bresser J. Kappelle L. J. Biessels G. J. Utrecht Diabetic Encephalopathy Study Group (2010). Cerebral cortical thickness in patients with type 2 diabetes. J. Neurol. Sci. 299, 126–130. 10.1016/j.jns.2010.08.048, PMID: 20869085

[ref7] Colcombe S. Kramer A. F. (2003). Fitness effects on the cognitive function of older adults: a meta-analytic study. Psychol. Sci. 14, 125–130. 10.1111/1467-9280.t01-1-01430, PMID: 12661673

[ref8] Conversano C. (2019). Common psychological factors in chronic diseases. Front. Psychol. 10:2727. 10.3389/fpsyg.2019.02727, PMID: 31866912 PMC6909152

[ref9] Cooke S. Pennington K. Jones A. Bridle C. Smith M. F. Curtis F. (2020). Effects of exercise, cognitive, and dual-task interventions on cognition in type 2 diabetes mellitus: a systematic review and meta-analysis. PLoS One 15:e0232958. 10.1371/journal.pone.0232958, PMID: 32407347 PMC7224461

[ref10] Crisafulli A. (2017). The impact of cardiovascular diseases on cardiovascular regulation during exercise in humans: studies on metaboreflex activation elicited by the post-exercise muscle ischemia method. Curr. Cardiol. Rev. 13, 293–300. 10.2174/1573403X13666170804165928, PMID: 28782491 PMC5730962

[ref11] Damasio A. Carvalho G. B. (2013). The nature of feelings: evolutionary and neurobiological origins. Nat. Rev. Neurosci. 14, 143–152. 10.1038/nrn3403, PMID: 23329161

[ref12] Delaney E. P. Greaney J. L. Edwards D. G. Rose W. C. Fadel P. J. Farquhar W. B. (2010). Exaggerated sympathetic and pressor responses to handgrip exercise in older hypertensive humans: role of the muscle metaboreflex. Am. J. Physiol. Heart Circ. Physiol. 299, H1318–H1327. 10.1152/ajpheart.00556.2010, PMID: 20802135 PMC2993192

[ref13] Doneddu A. Roberto S. Pinna V. Magnani S. Ghiani G. Sainas G.. (2020). Effect of combined mental task and metaboreflex activation on hemodynamics and cerebral oxygenation in patients with metabolic syndrome. Front. Physiol. 11:397. 10.3389/fphys.2020.00397, PMID: 32477157 PMC7241117

[ref14] Dupuy O. Gauthier C. J. Fraser S. A. Desjardins-Crepeau L. Desjardins M. Mekary S.. (2015). Higher levels of cardiovascular fitness are associated with better executive function and prefrontal oxygenation in younger and older women. Front. Hum. Neurosci. 9:66. 10.3389/fnhum.2015.00066, PMID: 25741267 PMC4332308

[ref15] Dyer A. H. Briggs R. Mockler D. Gibney J. Kennelly S. P. (2020). Non-pharmacological interventions for cognition in patients with type 2 diabetes mellitus: a systematic review. QJM: Int. J. Med. 113, 155–161. 10.1093/qjmed/hcz053, PMID: 30825309

[ref16] Esposito A. G. Baker-Ward L. Mueller S. (2013). Interference suppression vs. response inhibition: an explanation for the absence of a bilingual advantage in Preschoolers’ Stroop task performance. Cogn. Dev. 28, 354–363. 10.1016/j.cogdev.2013.09.002, PMID: 24453405 PMC3894626

[ref17] Fava A. Colica C. Plastino M. Messina D. Cristiano D. Opipari C.. (2017). Cognitive impairment is correlated with insulin resistance degree: the “PA-NICO-study”. Metab. Brain Dis. 32, 799–810. 10.1007/s11011-017-9977-4, PMID: 28229380

[ref18] Ferreri L. Bigand E. Perrey S. Bugaïska A. (2014). The promise of Near-Infrared Spectroscopy (NIRS) for psychological research: a brief review. L’Annee Psychologique 114, 537–569. 10.4074/s0003503314003054

[ref19] Gauvin L. Rejeski W. J. (1993). The exercise-induced feeling inventory: development and initial validation. J. Sport Exerc. Psychol. 15, 403–423. 10.1123/jsep.15.4.403

[ref20] González-Alonso J. Dalsgaard M. K. Osada T. Volianitis S. Dawson E. A. Yoshiga C. C.. (2004). Brain and central haemodynamics and oxygenation during maximal exercise in humans. J. Physiol. 557, 331–342. 10.1113/jphysiol.2004.060574, PMID: 15004212 PMC1665053

[ref21] Guicciardi M. Crisafulli A. Doneddu A. Fadda D. Lecis R. (2019). Effects of metabolic syndrome on cognitive performance of adults during exercise. Front. Psychol. 10:1845. 10.3389/fpsyg.2019.01845, PMID: 31440195 PMC6694762

[ref22] Guicciardi M. Lecis R. Anziani C. Corgiolu L. Porru A. Pusceddu M. (2014a). Type 2 diabetes: negative thoughts to physical activity. Sport Sci. Health 10, 247–251. 10.1007/s11332-014-0201-1 PMC4346010 25750816

[ref23] Guicciardi M. Lecis R. Anziani C. Corgiolu L. Porru A. Pusceddu M.. (2014b). Type 2 diabetes mellitus, physical activity, exercise self-efficacy, and body satisfaction. An application of the transtheoretical model in older adults. Health Psychol. Behav. Med. 2, 748–758. 10.1080/21642850.2014.924858, PMID: 25750816 PMC4346010

[ref24] Guicciardi M. Lecis R. Massidda D. Corgiolu L. Porru A. Pusceddu M. (2015). Inter-individual variability in psychological outcomes of supervised exercise in adults with type 2 diabetes/Variabilidad interindividual en los efectos psicológicos del ejercicio supervisado en adultos con diabetes tipo 2. Revista Costarricense de Psicología 34, 57–69. 10.22544/rcps.v34i02.01

[ref25] Hartman M. E. Ekkekakis P. Dicks N. D. Pettitt R. W. (2019). Dynamics of pleasure-displeasure at the limit of exercise tolerance: conceptualizing the sense of exertional physical fatigue as an affective response. J. Exp. Biol. 222:jeb186585. 10.1242/jeb.186585, PMID: 30559299

[ref26] Holtzer R. George C. J. Izzetoglu M. Wang C. (2018). The effect of diabetes on prefrontal cortex activation patterns during active walking in older adults. Brain Cogn. 125, 14–22. 10.1016/j.bandc.2018.03.002, PMID: 29807266 PMC6077094

[ref27] International Diabetes Federation (2017). International diabetes federation. Recommendations for managing type 2 diabetes in primary care. Available at: www.idf.org/managing-type2-diabetes (Accessed September 11, 2020).

[ref28] Kendzierski D. Johnson W. (1993). Excuses, excuses, excuses: a cognitive behavioral approach to exercise implementation. J. Sport Exerc. Psychol. 15, 207–219. 10.1123/jsep.15.2.207

[ref29] Kim C. Hwang A. Yoo J. (2004). The impact of a stage-matched intervention to promote exercise behaviour in participants with type 2 diabetes. Int. J. Nurs. Stud. 41, 833–841. 10.1016/j.ijnurstu.2004.03.009, PMID: 15476756

[ref30] Kim S.-H. Kim M. Ahn Y.-B. Lim H.-K. Kang S.-G. Cho J.-H.. (2011). Effect of dance exercise on cognitive function in elderly patients with metabolic syndrome: a pilot study. J. Sports Sci. Med. 10, 671–678. PMID: 24149557 PMC3761497

[ref31] Kim Y.-S. Seifert T. Brassard P. Rasmussen P. Vaag A. Nielsen H. B.. (2015). Impaired cerebral blood flow and oxygenation during exercise in type 2 diabetic patients. Physiol. Rep. 3:e12430. 10.14814/phy2.12430, PMID: 26109188 PMC4510631

[ref32] Kirk A. MacMillan F. Webster N. (2010). Application of the transtheoretical model to physical activity in older adults with type 2 diabetes and/or cardiovascular disease. Psychol. Sport Exerc. 11, 320–324. 10.1016/j.psychsport.2010.03.001

[ref33] Liu-Ambrose T. Nagamatsu L. S. Voss M. W. Khan K. M. Handy T. C. (2012). Resistance training and functional plasticity of the aging brain: a 12-month randomized controlled trial. Neurobiol. Aging 33, 1690–1698. 10.1016/j.neurobiolaging.2011.05.010, PMID: 21741129

[ref34] Lunghi C. Daniele G. Binda P. Dardano A. Ceccarini G. Santini F.. (2019a). Altered visual plasticity in morbidly obese subjects. iScience 22, 206–213. 10.1016/j.isci.2019.11.027, PMID: 31785558 PMC6909220

[ref35] Lunghi C. Sale A. (2015). A cycling lane for brain rewiring. Curr. Biol. 25, R1122–R1123. 10.1016/j.cub.2015.10.026, PMID: 26654367 PMC5040496

[ref36] Lunghi C. Sframeli A. T. Lepri A. Lepri M. Lisi D. Sale A.. (2019b). A new counterintuitive training for adult amblyopia. Ann. Clin. Transl. Neurol. 6, 274–284. 10.1002/acn3.698, PMID: 30847360 PMC6389748

[ref37] Lyu F. Wu D. Wei C. Wu A. (2020). Vascular cognitive impairment and dementia in type 2 diabetes mellitus: an overview. Life Sci. 254:117771. 10.1016/j.lfs.2020.117771, PMID: 32437791

[ref38] Manschot S. M. Biessels G. J. de Valk H. Algra A. Rutten G. E. H. M. van der Grond J.. (2007). Metabolic and vascular determinants of impaired cognitive performance and abnormalities on brain magnetic resonance imaging in patients with type 2 diabetes. Diabetologia 50, 2388–2397. 10.1007/s00125-007-0792-z, PMID: 17764005 PMC2039826

[ref39] Marcus B. H. Rossi J. S. Selby V. C. Niaura R. S. Abrams D. B. (1992). The stages and processes of exercise adoption and maintenance in a worksite sample. Health Psychol. 11, 386–395. 10.1037/0278-6133.11.6.386, PMID: 1286658

[ref40] Martino G. Caputo A. Bellone F. Quattropani M. C. Vicario C. M. (2020). Going beyond the visible in type 2 diabetes mellitus: defense mechanisms and their associations with depression and health-related quality of life. Front. Psychol. 11:267. 10.3389/fpsyg.2020.00267, PMID: 32174865 PMC7054284

[ref41] Martino G. Catalano A. Bellone F. Russo G. T. Vicario C. M. Lasco A.. (2019). As time goes by: anxiety negatively affects the perceived quality of life in patients with type 2 diabetes of long duration. Front. Psychol. 10:1779. 10.3389/fpsyg.2019.01779, PMID: 31428028 PMC6689992

[ref42] Masters K. Spielmans G. LaCaille R. Goodson J. Larsen B. Heath E. (2011). Effects of home exercise on immediate and delayed affect and mood among rural individuals at risk for type 2 diabetes. J. Soc. Behav. Health Sci. 5:1. 10.5590/JSBHS.2011.05.1.01

[ref43] Olson E. A. Mullen S. P. Raine L. B. Kramer A. F. Hillman C. H. McAuley E. (2017). Integrated social‐ and neurocognitive model of physical activity behavior in older adults with metabolic disease. Ann. Behav. Med. 51, 272–281. 10.1007/s12160-016-9850-4, PMID: 27844326 PMC5475366

[ref44] Palta P. Schneider A. L. C. Biessels G. J. Touradji P. Hill-Briggs F. (2014). Magnitude of cognitive dysfunction in adults with type 2 diabetes: a meta-analysis of six cognitive domains and the most frequently reported neuropsychological tests within domains. J. Int. Neuropsychol. Soc. 20, 278–291. 10.1017/S1355617713001483, PMID: 24555960 PMC4132660

[ref45] Plichta M. M. Herrmann M. J. Ehlis A. -C. Baehne C. G. Richter M. M. Fallgatter A. J. (2006). Event-related visual versus blocked motor task: detection of specific cortical activation patterns with functional near-infrared spectroscopy. Neuropsychobiology 53, 77–82. 10.1159/000091723, PMID: 16511338

[ref46] Podolski N. Brixius K. Predel H. G. Brinkmann C. (2017). Effects of regular physical activity on the cognitive performance of type 2 diabetic patients: a systematic review. Metab. Syndr. Relat. Disord. 15, 481–493. 10.1089/met.2017.0120, PMID: 29160740

[ref47] Rasmussen P. Nielsen J. Overgaard M. Krogh-Madsen R. Gjedde A. Secher N. H.. (2010). Reduced muscle activation during exercise related to brain oxygenation and metabolism in humans. J. Physiol. 588, 1985–1995. 10.1113/jphysiol.2009.186767, PMID: 20403976 PMC2901984

[ref48] Rooks C. R. Thom N. J. McCully K. K. Dishman R. K. (2010). Effects of incremental exercise on cerebral oxygenation measured by near-infrared spectroscopy: a systematic review. Prog. Neurobiol. 92, 134–150. 10.1016/j.pneurobio.2010.06.002, PMID: 20542078

[ref49] Sadanand S. Balachandar R. Bharath S. (2016). Memory and executive functions in persons with type 2 diabetes: a meta-analysis. Diabetes Metab. Res. Rev. 32, 132–142. 10.1002/dmrr.2664, PMID: 25963303

[ref50] Secher N. H. Seifert T. Van Lieshout J. J. (2008). Cerebral blood flow and metabolism during exercise: implications for fatigue. J. Appl. Physiol. 104, 306–314. 10.1152/japplphysiol.00853.2007, PMID: 17962575

[ref51] Settineri S. Frisone F. Merlo E. M. Geraci D. Martino G. (2019). Compliance, adherence, concordance, empowerment, and self-management: five words to manifest a relational maladjustment in diabetes. J. Multidiscip. Healthc. 12, 299–314. 10.2147/JMDH.S193752, PMID: 31118655 PMC6499139

[ref52] Strangman G. Boas D. A. Sutton J. P. (2002). Non-invasive neuroimaging using near-infrared light. Biol. Psychiatry 52, 679–693. 10.1016/S0006-3223(02)01550-0, PMID: 12372658

[ref53] Sun L. Diao X. Gang X. Lv Y. Zhao X. Yang S.. (2020). Risk factors for cognitive impairment in patients with type 2 diabetes. J. Diabetes Res. 2020, 1–10. 10.1155/2020/4591938, PMID: 32377520 PMC7196145

[ref54] Terada T. Friesen A. Chahal B. S. Bell G. J. McCargar L. J. Boulé N. G. (2013). Feasibility and preliminary efficacy of high intensity interval training in type 2 diabetes. Diabetes Res. Clin. Pract. 99, 120–129. 10.1016/j.diabres.2012.10.019, PMID: 23183390

[ref55] Tomas-Carus P. Ortega-Alonso A. Pietilainen K. H. Santos V. Goncalves H. Ramos J.. (2016). A randomized controlled trial on the effects of combined aerobic-resistance exercise on muscle strength and fatigue, glycemic control and health-related quality of life of type 2 diabetes patients. J. Sports Med. Phys. Fitness 56, 572–578. PMID: 27285345

[ref56] Tuomiletho J. Lindstrom J. Eriksson J. G. Valle T. T. Hamalainen H. Ilanne-Parikka P.. (2001). Prevention of type 2 diabetes mellitus by changes in lifestyle among subjects with impaired glucose tolerance. N. Engl. J. Med. 344, 1343–1350. 10.1056/NEJM200105033441801, PMID: 11333990

[ref57] van den Berg E. Kloppenborg R. P. Kessels R. P. C. Kappelle L. J. Biessels G. J. (2009). Type 2 diabetes mellitus, hypertension, dyslipidemia and obesity: a systematic comparison of their impact on cognition. Biochim. Biophys. Acta 1792, 470–481. 10.1016/j.bbadis.2008.09.004, PMID: 18848880

[ref58] van Elderen S. G. C. de Roos A. de Craen A. J. M. Westendorp R. G. J. Blauw G. J. Jukema J. W.. (2010). Progression of brain atrophy and cognitive decline in diabetes mellitus: a 3-year follow-up. Neurology 75, 997–1002. 10.1212/WNL.0b013e3181f25f06, PMID: 20837967

[ref59] Vianna L. C. Deo S. H. Jensen A. K. Holwerda S. W. Zimmerman M. C. Fadel P. J. (2015). Impaired dynamic cerebral autoregulation at rest and during isometric exercise in type 2 diabetes patients. Am. J. Physiol. Heart Circ. Physiol. 308, H681–H687. 10.1152/ajpheart.00343.2014, PMID: 25599569 PMC4385994

[ref60] Zarahn E. Rakitin B. Abela D. Flynn J. Stern Y. (2007). Age-related changes in brain activation during a delayed item recognition task. Neurobiol. Aging 28, 784–798. 10.1016/j.neurobiolaging.2006.03.002, PMID: 16621168

[ref61] Zhao Q. Zhang Y. Liao X. Wang W. (2020). Executive function and diabetes: a clinical neuropsychology perspective. Front. Psychol. 11:2112. 10.3389/fpsyg.2020.02112, PMID: 32973635 PMC7468478

[ref62] Zhao R. R. O’Sullivan A. J. Fiatarone Singh M. A. (2018). Exercise or physical activity and cognitive function in adults with type 2 diabetes, insulin resistance or impaired glucose tolerance: a systematic review. Eur. Rev. Aging Phys. Act. 15:1. 10.1186/s11556-018-0190-1, PMID: 29387262 PMC5776769

