# Supplementary material for: Affective Variables and Cognitive Performances During Exercise in a Group of Adults With Type 2 Diabetes Mellitus
Source: Front Psychol. 2020 Dec 23;11:611558. doi: 10.3389/fpsyg.2020.611558 (PMC7785934; doi:10.3389/fpsyg.2020.611558)

Supplementary Material

1 Supplementary Table

Table 1 Spearman's correlations between sessions and biomedical variables

	BST	CER+BST	PEMI+BST	AGE	BMI	YEARS SINCE DIAGNOSIS
BST	1					
CER+BST	.664*	1				
PEMI+BST	.769**	.755**	1			
AGE	.233	.190	.487	1		
BMI	-.280	-.245	-.091	-.049	1	
YEARS SINCE DIAGNOSIS	.453	.496	.485	.172	.244	1

* p < .05

** p < .01

Supplementary Figures

Fig.1 Scatterplot of age and reaction times by sessions

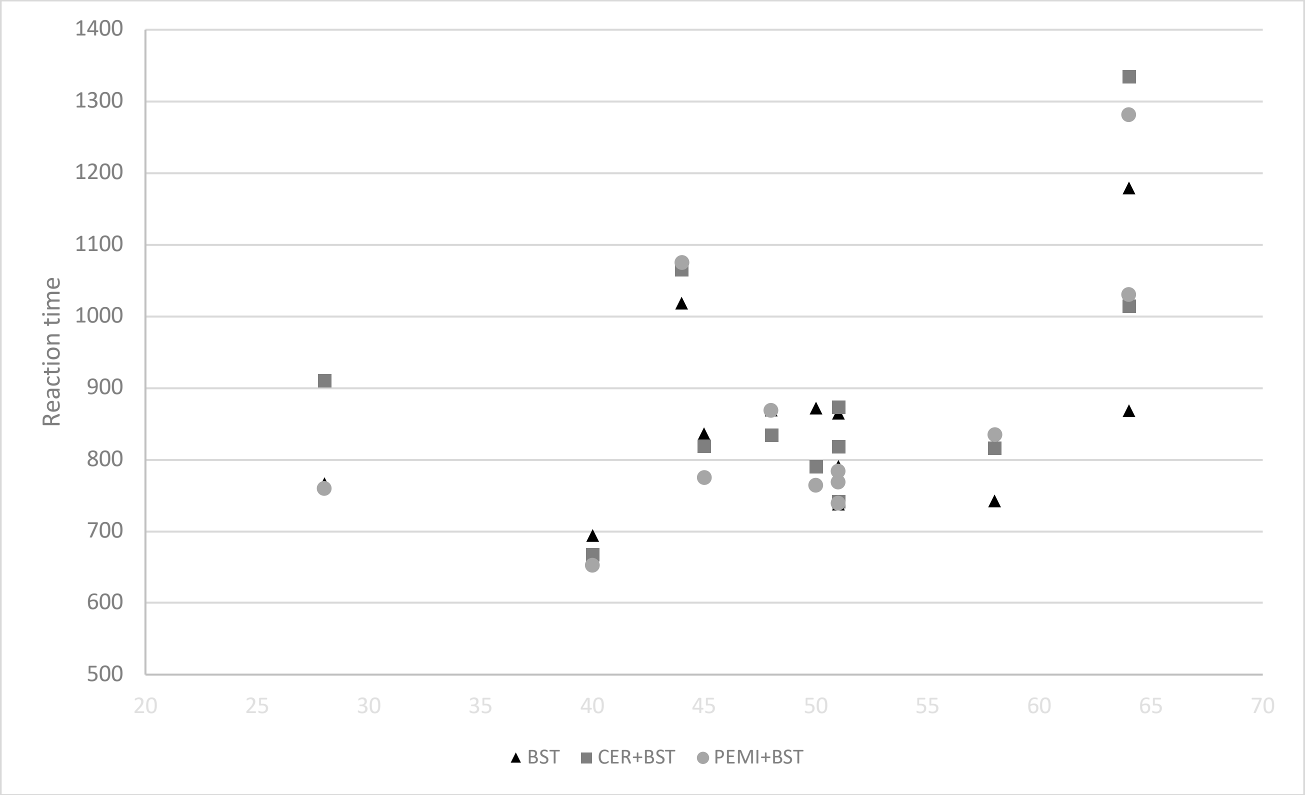

Fig. 2 Scatterplot of BMI and rection times by sessions

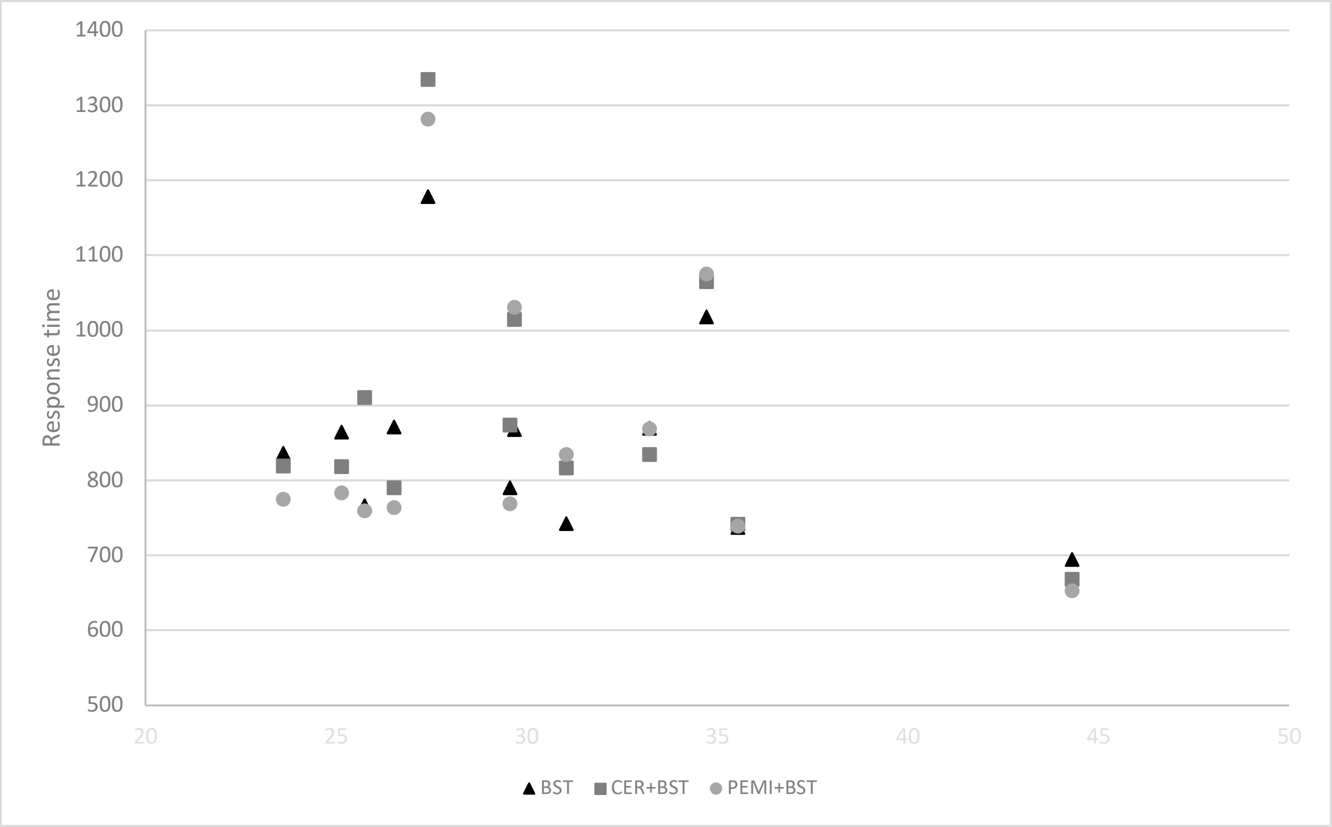

Supplement: Supplementary file 1 [file Data_Sheet_1.pdf]
